# Homogenized gridded dataset for drought and hydrometeorological modeling for the continental United States

**DOI:** 10.1038/s41597-024-03202-6

**Published:** 2024-04-12

**Authors:** Robert Erhardt, Courtney A. Di Vittorio, Staci A. Hepler, Lauren E. L. Lowman, Wendy Wei

**Affiliations:** 1 Wake Forest University, Department of Statistical Sciences, Winston-Salem, NC USA; 2 Wake Forest University, Department of Engineering, Winston-Salem, NC USA

**Keywords:** Environmental sciences, Natural hazards

## Abstract

We present a novel data set for drought in the continental US (CONUS) built to enable computationally efficient spatio-temporal statistical and probabilistic models of drought. We converted drought data obtained from the widely-used US Drought Monitor (USDM) from its native geo-referenced polygon format to a 0.5 degree regular grid. We merged known environmental drivers of drought, including those obtained from the North American Land Data Assimilation System (NLDAS-2), US Geological Survey (USGS) streamflow data, and National Oceanic and Atmospheric Administration (NOAA) teleconnections data. The resulting data set permits statistical and probabilistic modeling of drought with explicit spatial and/or temporal dependence. Such models could be used to forecast drought at short-range, seasonal to sub-seasonal, and inter-annual timescales with uncertainty, extending the reach and value of the current US Drought Outlook from the National Weather Service Climate Prediction Center. This novel data product provides the first common gridded dataset that includes critical variables used to inform hydrological and meteorological drought.

## Background & Summary

The last two decades have seen strong development of gridded drought indices. The global standardized precipitation evapotranspiration index database (SPEIBASE) was first released in 2010^1,2^. It utilizes gridded public climate data available monthly at 0.5 degrees and constructs a drought index based on both precipitation as well as evapotranspiration. The Global Precipitation Climatology Centre Drought Index (GPCC-DI) extended this to produce an index which combined a standardized precipitation index (SPI) with the SPEI to achieve a new global, monthly gridded drought index, but at a lower 1 degree spatial resolution^3^. Several researchers have achieved higher spatial resolutions in regional data products. Two examples include a combined SPI/SPEI index achieved daily at 0.1 degrees in China^4^, and a suite of drought indices at the high 12 km resolution produced over a small region covering three states in the United States^5^.

Enhancements described above include new data sources, superior spatio-temporal resolutions, and region-specific data sets more suited to studying local drought. Here we present a drought database for the continental United States that has all three enhancements. That is, this database: includes an enhanced drought index which draws on both high quality environmental data sources as well as expert local regional judgments; includes a suite of hydrometeorological variables which permits scientific study of regional drought processes across a range of scales; and achieves a superior spatio-temporal resolution than what is currently available from global products which cover the contintental United States. We achieve these goals by constructing a gridded drought database from the U.S. Drought Monitor (USDM), and merging in a suite of hydrometeorological variables at the same spatio-temporal resolution.

**Fig. 1:**
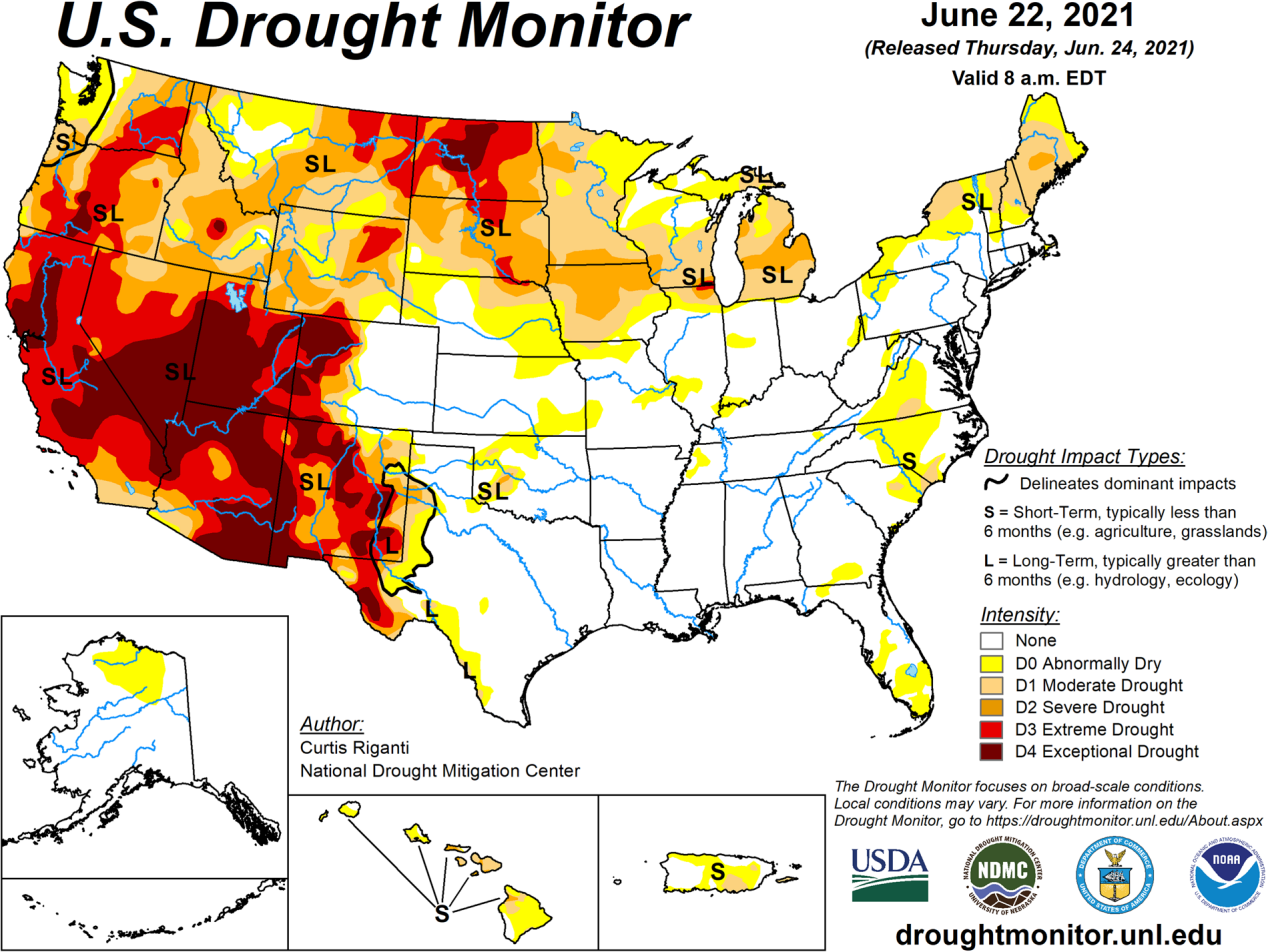
An example of the raw data for the U.S. Drought Monitor for June 22, 2021. The U.S. Drought Monitor is jointly produced by the National Drought Mitigation Center at the University of Nebraska-Lincoln, the United States Department of Agriculture, and the National Oceanic and Atmospheric Administration. Map courtesy of NDMC.

The U.S. Drought Monitor (USDM)^6^ provides a weekly snapshot of the drought status in the United States. The U.S. Drought Monitor is jointly produced by the National Drought Mitigation (NDMC) at the University of Nebraska-Lincoln, the United States Department of Agriculture (USDA), and the National Oceanic and Atmospheric Administration (NOAA). This data product was first released on January 4, 2000, and has been updated weekly ever since. It characterizes drought severity as of 8AM EST/EDT each Tuesday for the entire U.S., though the images are released two days later on the following Thursday. The monitor classifies drought into one of six ordered categories: 0, no drought; D0, abnormally dry or “pre-drought”; and levels D1 through D4, which represent increasing levels of drought severity. Figure 1 shows the USDM as of 8AM EST on June 22, 2021. The USDM is defined everywhere in space over the contiguous United States, and also Alaska, Hawaii, Puerto Rico, and some outlying territories.

The classification of drought into the ordinal measure relies on inputs such as the Palmer Drought Severity Index^7^, soil moisture^8^, United States Geological Survey weekly streamflow data (https://waterwatch.usgs.gov/), and other environmental inputs. However, the USDM is *not* a deterministic calculation based on these inputs. Rather, the NDMC website describes the USDM as a blending of the “best available data, local observations, and experts’ best judgment that makes the U.S. Drought Monitor more versatile than other drought indicators. (https://droughtmonitor.unl.edu/About/AbouttheData/DroughtClassification.aspx)”.

**Fig. 2:**
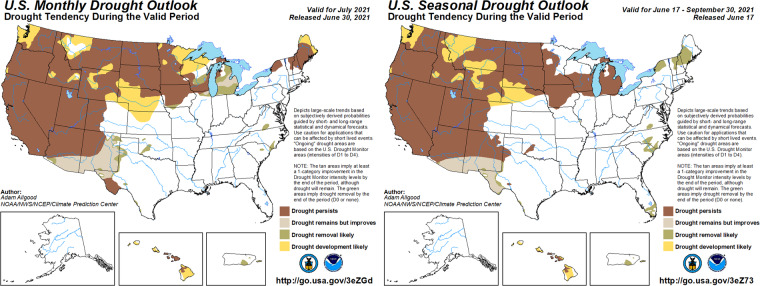
Examples of a monthly US Drought Outlook (left) and seasonal US Drought Outlook (right) released in June 2021, around the same time as the USDM shown in Fig. 1 was released. No quantification of forecast uncertainty is captured or conveyed.

USDM images are updated each week, and this is used to define the weekly time scale for our data product described in this paper. The USDM images are freely available as shapefiles (https://droughtmonitor.unl.edu/DmData/GISData.aspx). While this data structure is ideal for visualizing drought status continuously in space, it presents challenges for building statistical models to answer scientific questions. Examples of such questions include:

- How much variability in drought level can be explained by uncertainty in meteorological and land-surface conditions? In contrast, how much variability is explained by “local observations and experts’ best judgment”?
- What is the relative importance of each individual USDM input to the overall estimated drought level? How much value would a proposed new scientific input add? How does the relative importance of individual inputs vary across space and time?
- How can we best make projections of drought status into the future? The current drought forecast tool is the US Drought Outlook, published each month and shows expectations of future droughts one month and one season ahead (Fig. 2). While informative for many purposes, the US Drought Outlook is limited in that it does not capture or convey forecast uncertainty. How could such forecasts be enhanced to quantify uncertainty across space and time?

To answer any of the previous questions, one would need a statistical model relating the ordinal response of drought level to other covariates. This immediately raises a question–what should the spatio-temporal support be for such a model? The response variable of drought level is defined everywhere in space, stored as geo-referenced polygons, and therefore not easily used as a response variable in its native form. On the other hand, the environmental covariates are available at different spatial and temporal resolutions. Some–such as USGS streamflow–are point-referenced and obtained at specific locations sampled unevenly and irregularly across the US. Others–such as meteorological variables–are provided as satellite or reanalysis products stored on a regular grid indexed by latitude and longitude. Modeling the USDM drought severity in a statistical framework therefore necessarily blends vectorized images, point-referenced data with incomplete coverage, and gridded aerial observations, all sampled at different time scales. Our solution presented here is to process all available data to a common spatio-temporal support.

With an eye towards scientific questions involving forecasting, we consider additional covariates beyond those used to define the USDM. One study used predictors obtained from the North American Multi-Model Ensemble (NMME)^9^ climate forecasts to produce 1- and 3-month forecasts of the USDM^10^. They noted that more environmental variables beyond precipitation and temperature are used to construct the USDM, and therefore the NMME dataset should be supplemented with other climate information to produce the best forecasts. In our data product, we added accumulated precipitation, total evapotranspiration, potential evapotranspiration, potential latent heat flux, soil moisture, surface runoff, soil temperature, leaf area index, snow depth, snow melt, snow cover fraction, and water equivalent of accumulated snow depth. Numerous studies have also established the importance of using the El Niño Southern Oscillation (ENSO) index for drought modeling^10–18^. Other studies have considered climate teleconnections such as the North Atlantic Oscillation (NAO)^19^. Accordingly, we added four teleconnections to our data product. These teleconnections are notably different from the other hydrometeorological and hydrological drought-forcing variables in a number of ways. First, they are (at most) statistically related to drought, rather than directly and locally-acting as the other variables. Second, users should likely consider the teleconnections as statistically-linked to drought with spatio-temporally-varying coefficients, which would allow the statistical relationship between the teleconnection to vary in space and/or time. Users could also include subsets of teleconnections in a hierarchical model, using them as covariates for hydrometeorological and hydrological variables in a middle layer of model, leaving the hydrometeorological and hydrological variables alone to directly act on drought. Regardless of the particular use, the point is that teleconnections differ from the other locally-varying and direct variables in these data.

**Fig. 3:**
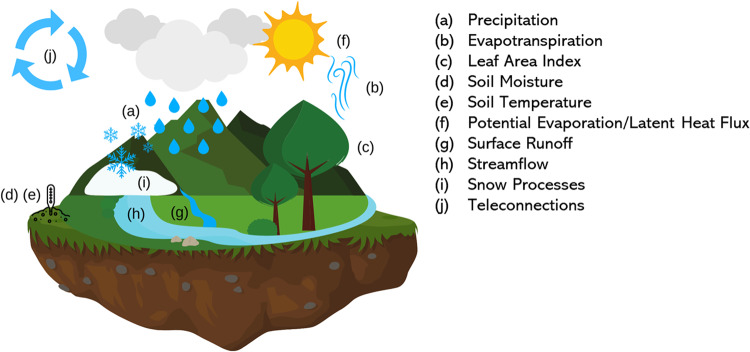
Summary of climate, atmospheric, and land surface variables included in the homogenized dataset. All variables have been demonstrated in the literature to be informative of drought and/or its impacts.

It is clear that there is a scientific need for a database to support statistical research using the USDM as well as seasonal to sub-seasonal (S2S) forecasting of drought, where predictive skill is relatively low^20^. This new database should include known scientific inputs for the USDM along with other potential predictors of drought (Fig. 3). All data should be recorded at the same spatial and temporal support to avoid the need for change of support models which could otherwise increase the computational cost of fitting models. In this paper, we describe the construction of a comprehensive database which discretizes the USDM to a 0.5 degree spatial resolution, and merges in summaries of NLDAS-2 data and USGS streamflow data along with common climate teleconnections, all at the same spatial resolution and weekly time scale (see Tables 1 and 2).

**Table 1:** Data sources for all NLDAS-2 climate variables combined with USDM data.

Data type	Description	Time	Units	Spatial resolution	Temporal resolution
Precipitation^30,66^	Hourly total accumulated precipitation calculated using gauge-only daily precipitation analyzed by NCEP’s Climate Prediction Center. NLDAS-2 Forcing file A	01/01/2000-06/30/2022	kg m^−2^	0.125°	1-hour
Potential Evapotranspiration^67,68^	Evaporation occurring over a free water surface assuming unlimited water supply and accounting for aerodynamic resistance and the surface energy budget. NLDAS-2 Forcing file A	01/01/2000-06/30/2022	kg m^−2^	0.125°	1-hour
Total Evapotranspiration^31,69^	Total land evapotranspiration as the sum of evaporation from bare soil, canopy interception, and transpiration from plants. NLDAS-2 Noah LSM	01/01/2000-06/30/2022	kg m^−2^	0.125°	1-hour
Potential Latent Heat Flux^30,66^	Potential evapotranspiration in energy units calculated as the sum of evaporation from bare soil and canopy interception, transpiration from the canopy, and sublimation from snowpack. NLDAS-2 Noah LSM	01/01/2000-06/30/2022	W m^−2^	0.125°	1-hour
Soil Moisture Content^30,31,66,70^	Amount of water held in 4 distinct soil layers (0–10 cm, 10–40 cm, 40–100, and 100–200 cm) estimated from the Noah land-surface model using coupled energy and water balances. NLDAS-2 Noah LSM	01/01/2000-06/30/2022	kg m^−2^	0.125°	1-hour
Surface Runoff^30,31,66,70^	Accumulated liquid water on the land surface from precipitation that is not infiltrated into soils. NLDAS-2 Noah LSM	01/01/2000-06/30/2022	kg m^−2^	0.125°	1-hour
Soil Temperature^30,31,66,71^	Temperature of 4 distinct soil layers (0–10 cm, 10–40 cm, 40–100 cm, and 100–200 cm) estimated from the Noah land-surface model. NLDAS-2 Noah LSM	01/01/2000-06/30/2022	K	0.125°	1-hour
Leaf Area Index^30,31,66^	Unitless parameter that quantifies vegetation canopy density as the ratio the one-sided area of leaf material in the canopy per unit ground area. NLDAS-2 Noah LSM	01/01/2000-06/30/2022	unitless	0.125°	1-hour
Snow Depth^33,72^	Instantaneous depth of snow accumulated on the land surface estimated from the Noah land-surface model. NLDAS-2 Noah LSM	01/01/2000-06/30/2022	m	0.125°	1-hour
Snow Melt^33,72^	Instantaneous amount of liquid water produced by snow melt on the land surface estimated from the Noah land-surface model. NLDAS-2 Noah LSM	01/01/2000-06/30/2022	kg m^−2^	0.125°	1-hour
Snow Cover^33,72^	Fraction of land pixel covered with snow computed as a non-linear function of snow water equivalent (SWE) utilizing a generalized snow depletion curve. NLDAS-2 Noah LSM	01/01/2000-06/30/2022	unitless	0.125°	1-hour
Water Equivalent of Accumulated Snow Depth^33,72^	Equivalent amount of liquid water stored in snowpack estimated from the Noah land-surface model. NLDAS-2 Noah LSM	01/01/2000-06/30/2022	kg m^−2^	0.125°	1-hour

**Table 2:** Data sources for all streamflow and teleconnections climate variables combined with USDM data.

Data type	Description	Time	Units	Spatial resolution	Temporal resolution
Streamflow (https://waterdata.usgs.gov/nwis/sw)	In-site streamflow measurements obtained fromUSGS gauges	*m*^3^/*s*	point	daily	
Pacific North American Pattern	A pattern of air pressure anomalies over the Pacific Ocean and North America	01/01/1950 - present	unitless	none	daily
North Atlantic Oscillation^73^	An atmospheric phenomena related to the difference in pressure at sea level between Iceland and the Azores High	01/01/1950 - present	unitless	none	daily
Arctic Oscillation^74–77^	An index computed from differences in sea-level pressure between anomalies in the Arctic and anomalies around 37–45° N	01/01/1950 - present	unitless	none	daily
El Niño Southern Oscillation^78^	An index computed from sea-surface temperature anomalies in a region off the coast of South America. Included in 7-day, 14-day, 29-day, and 84-day averages.	09/02/1981 - present	unitless	none	weekly

## Methods

**Fig. 4:**
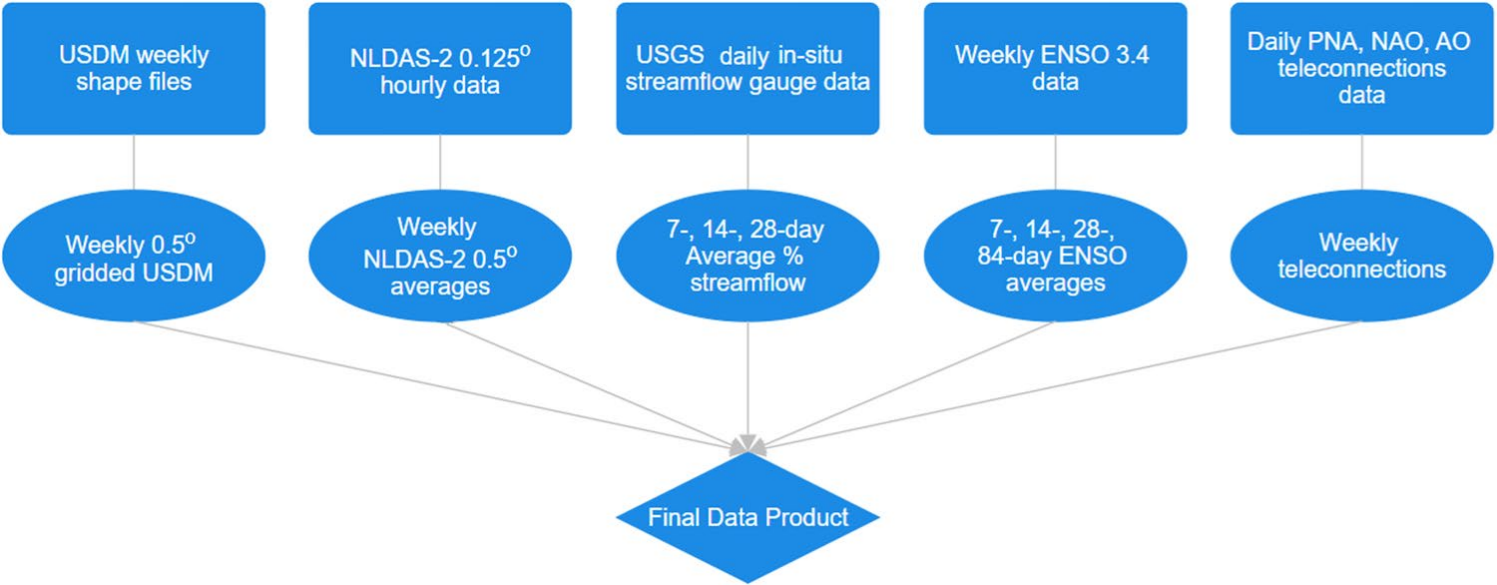
Flowchart of the processing of raw data sources to a combined final data product.

We process and combine four sources of raw data: USDM drought data, NLDAS-2 hydrometeorological data, USGS streamflow data, and teleconnections data. Figure 4 shows this process, each component of which is described below.

### US Drought Monitor

US Drought Monitor raw data are available as geo-referenced polygons stored as shapefiles, released each week. One example is shown in Fig. 1. The primary processing step consisted of discretizing these weekly geo-referenced polygons to a common gridded support covering the contiguous United States. We defined a regular grid whose centroids are a set of points evenly spaced every 0.5 degrees by both latitude and longitude, and this grid serves as the common spatial support for all variables in the data set. Numerous studies have used gridded drought data at various scales, including 1 degree^3,21^, 0.5 degree^22^, and 0.3 degrees^23,24^. Studies analyzing drought in a statistical or probabilistic framework have used spatial resolutions of 0.125 degrees^25,26^, 0.25 degrees^27^, 0.5 degrees^11,28,29^, or 1 degree^10^, depending on the spatial resolutions of the particular predictor variables.

**Fig. 5:**
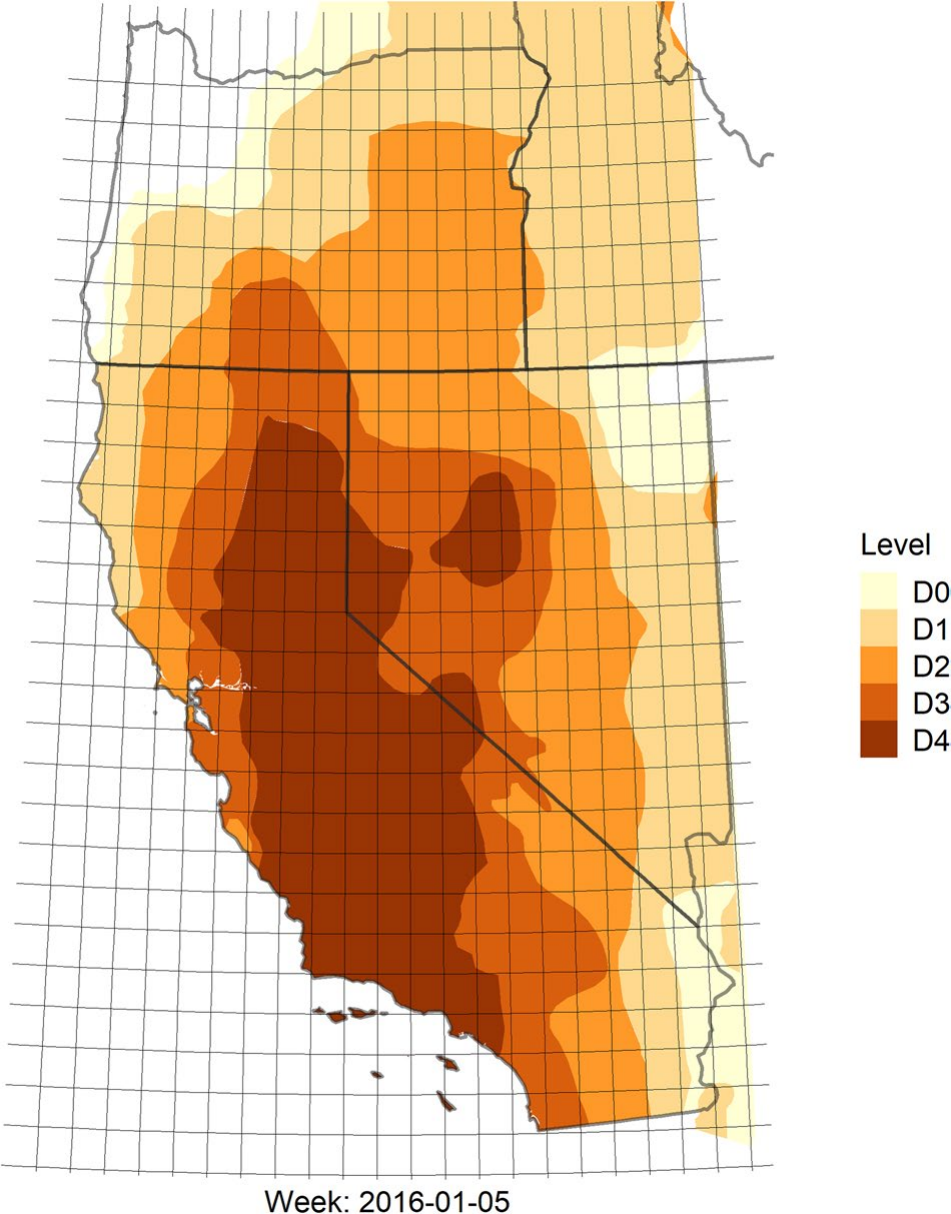
Overlay of the 0.5 degree grid over a portion of the western United States, showing USDM values for 01/05/2016. Our method selected the centroid of each grid cell. Other regions and times demonstrate a similar correspondence between the centroid and the mode (computed spatially).

**Fig. 6:**
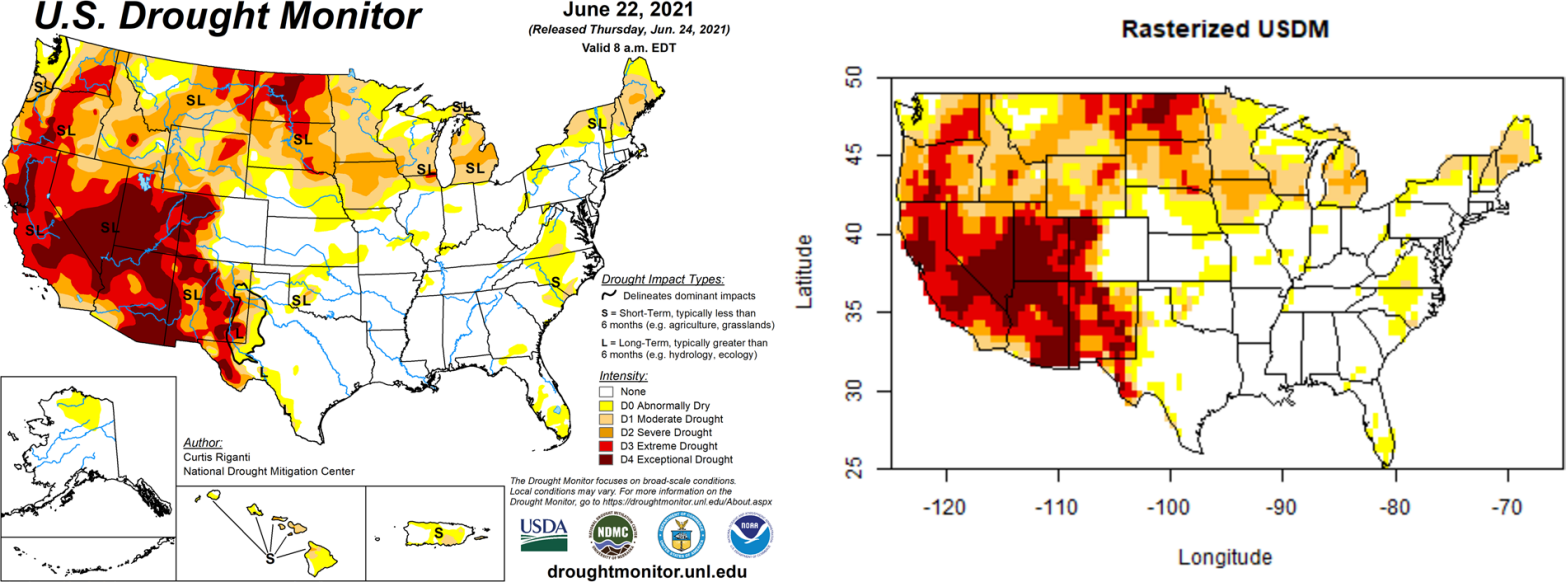
Left panel: Raw data for the U.S. Drought Monitor for June 22, 2021. Right panel: Discretized data from the same time period on a 0.5 degree spatial support of 3258 grid cells covering the contiguous US only.

We began with a bounding box which ranged from −124.75 degrees longitude to −65.25 degrees longitude, and 25.25 degrees latitude to 49.75 degrees latitude. Grid cells were labeled with the letter referring to each of the 50 rows (latitude) from letters ordered as A,…, Z, AA,…, XX beginning at 49.75 degrees north, and columns with numbers from 1 to 120 beginning at −124.75 degrees west. All lat/lon coordinates are defined at the grid centroid. Thus, the bounding box is indexed by A1 (−124.75, 49.75) to XX120 (−65.25, 25.25). However, only 3259 of these 6000 grid cell locations fall within the contiguous United States over land where the USDM is defined. We will later see that one grid cell over Great Salt Lake is primarily water with undefined land variables, and so *i* = 1, …, *I* = 3258 will index spatial location in our data. Accordingly, the top-left grid that appears in the data product is C6 at (−122.25, 48.75), and the bottom-right is XX89 at (−80.75, 25.25). The particular value of the USDM is taken from the centroid of each grid cell, for each week–i.e. there is no averaging within a grid cell. This choice results in a gridded summary of drought that has the same support as the USDM itself (0, D0, D1,…, D4) and is therefore interpretable and familiar to all end users of the USDM (unlike, for example, averaging levels within a grid cell, which would produce values between the familiar support of the USDM). For grid cells with two or more levels of drought falling within a single cell, we explored taking the mode (by area within each cell), but found it made very little practical difference compared to the centroid, as the two values are identical in nearly every case. Figure 5 shows one example of the 0.5 degree grid overlaying USDM data in one region of the United States to demonstrate visually the consistency between the centroid and mode. Figure 6 shows the result of this discretization process for the week of June 22, 2021. The same process is applied to each week of the available USDM data, beginning 01/04/2000 (*t* = 1) and ending on 06/30/2022 (*t* = 1174). This results in the database having exactly 3,824,892 rows (3258 × 1174), each of which has columns indicating the grid name, latitude, longitude, time, and measurement of drought.

### NLDAS-2

The North America Land Data Assimilation System Phase 2 (NLDAS-2) is an integrated observation and model reanalysis data set designed to drive offline land surface models^30,31^. NLDAS-2 land surface forcing fields are derived from the North American Regional Reanalysis (NARR) fields and are available at 0.125° × 0.125° spatial resolution, and at hourly or monthly temporal resolutions. Specific details on the spatial interpolation and temporal disaggregation methods adopted in NLDAS-2 are described in Cosgrove *et al*.^32^. Accumulated precipitation [kg/m^2^] and potential evapotranspiration [kg/m^2^] were obtained as covariates for the statistical model from the NLDAS-2 Forcing File A dataset. These variables constrain water availability for a given time period. Outputs from the NLDAS Noah land-surface model forced with the forcing fields were also obtained as covariates. The Noah land-surface model performs a water and energy balance for the land-surface, which is discretized into four soil layers, and parameterizes warm and cold season processes^33,34^. The specific outputs used as covariates are total evapotranspiration [kg/m^2^], potential latent heat flux [W/m^2^], soil moisture from the top soil layer with a thickness of 10 cm [kg/m^2^], surface runoff [kg/m^2^], temperature of the top soil layer [K], leaf area index [-], snow depth [m], snow melt [kg/m^2^], snow cover fraction [-], and water equivalent of accumulated snow depth [kg/m^2^]. All datasets are freely available to download from NASA’s Goddard Earth Sciences Data and Information Services Center (GES DISC)(https://disc.gsfc.nasa.gov/).

We selected variables from the NLDAS-2 dataset that are strongly linked to hydrological and meteorological drought. Total evapotranspiration and potential latent heat flux provide estimates of how much water is leaving the land surface to the atmosphere, while surface runoff characterizes how much how is being transported out of a given grid cell. Soil moisture quantifies water storage within the grid cell, and soil temperature can indicate heat stress associated with drought. Leaf area index (LAI) describes current plant growth stage, which can be disrupted during severe drought events^35^. LAI predicted from the Noah land-surface model and is spatially and seasonally varying. It depends on land cover type and green vegetation cover fraction^34^. Snow depth, snow melt, snow cover fraction, and the water equivalent of accumulated snow depth are included as many regions in the Western US depend on the slow release of water from snow to sustain periods of low rainfall during the warm season. It is important to note that potential evapotranspiration and potential latent heat flux are essentially the same variable, as latent heat equals evapotranspiration multiplied by the latent heat of vaporization. One would only want to use at most one of these in a statistical model of these data, however we include both as different research communities have different preferences around which variable to use. Similary, snow depth and the water equivalent of accumulated snow depth provide similar information, and only one of these variables should be used in a statistical model.

**Fig. 7:**
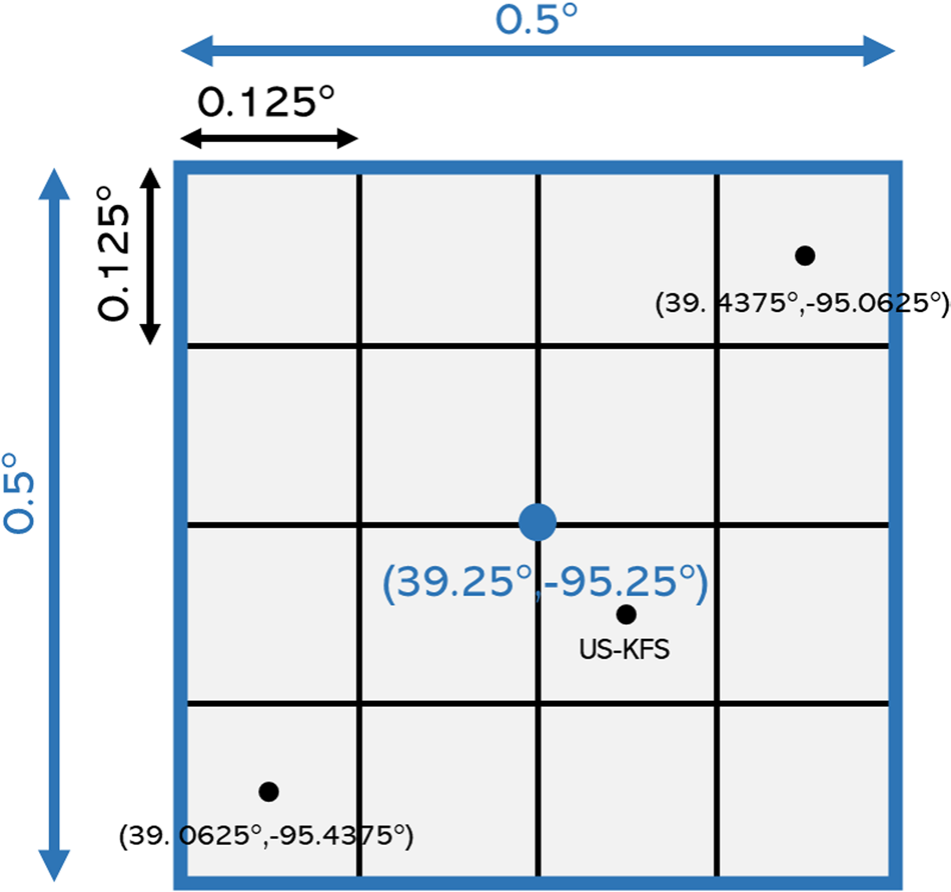
Graphical representation of the upscaling procedure for the NLDAS-2 data for pixels surrounding the US Kansas Field Station (US-KFS) (39.0561°, −95.1907°). Sixteen pixels from the 0.125° native resolution are aggregated to the 0.5° grid. Specifically, for all variables except for precipitation, the average of the 0.125° pixels is computed and taken as the value across the entire 0.5° pixel. The sum of all 16 pixels is used for precipitation.

Hourly NLDAS-2 Forcing File A and Noah land-surface model datasets were downloaded for all of CONUS between January 1, 2000 and June 30, 2022 from the NASA GES DISC. To upscale the data from 0.125° × 0.125° to the common 0.5° spatial support, 16 pixels from the native NLDAS-2 resolution were aggregated to the 0.5 resolution (Fig. 7). For variables that were derived from the Noah land-surface model, water pixels were masked out (as land processes are not valid to approximate over water and not produced by the Noah model). We required that at least 70% of the 0.125° pixels (or 11 pixels total) must be land pixels in order to define and compute an upscaled value at the 0.5° resolution; otherwise, the upscaled grid cell was designated as a water pixel at the coarser scale. Then, each dataset was aggregated to a weekly timestep that aligned with the USDM. For all variables except precipitation, the data were aggregated by taking the average in space and time. For precipitation, the sum is used to aggregate the data in space and in time to the 0.5° and weekly resolutions to aid with interpretability (https://climatedataguide.ucar.edu/climate-tools/regridding-overview).

### USGS Streamflow

Gridded data on observed streamflow is not readily available. Streamflow data is point-based and collected from gauges that are unequally distributed in space and have variable temporal coverage. Streamflow percentiles that represent flow magnitude relative to the historical gauge record have been used as a direct measure of hydrologic drought, where thresholds have been used to identify drought severity, duration, and frequency^36^ and the extent to which these characteristics have changed over time^37^. Long-term records of streamflow have been related to regional climate and hydrologic processes^38^, indicating that this information could also inform more holistic drought assessments, such as the USDM drought severity metric. Many state-level drought advisory councils currently use point-based streamflow percentiles in their manual assessments that inform the USDM^39,40^.

We derived 7-day, 14-day, and 28-day streamflow percentiles for each *in-situ* gauge to be consistent with how state-level drought advisory councils manually aggregate streamflow data to inform the USDM^39,40^. Using weekly to monthly averages allows for the incorporation of hydrologic processes that impact drought on different times scales; for example, groundwater infiltration resulting from a storm event is a very slow process compared to surface water runoff. The percentiles represent the magnitude of flow relative to what has been observed in the past and are used to assess drought severity, where areas with lower percentiles are often interpreted to have higher drought risk. Using this relative measure instead of an absolute measure helps managers combine and directly compare streamflow gauges with a wide range of flow magnitudes, enabling drought assessments on large spatial scales. To produce our gridded streamflow percentiles data product, we used the following data processing workflow broadly summarized as follows (and described in greater detail in the subsequent text, tables, and figures):

1. We downloaded all CONUS-scale *in-situ* streamflow data and identified the gauges that fall within each model grid.
2. We calculated the 7-day, 14-day, and 28-day streamflow averages as of each weekly time period in the USDM dataset, and converted each observation from flow (cfs) to a percentile based on its rank within a baseline empirical distribution. All streamflow observations from 1990 to 2020 were used to create the baseline distribution for each gauge. This 30-year time period is consistent with the World Meteorological Organization climatology standard normals.
3. For all grid cells and dates that did contain *in-situ* data, we calculated the arithmetic means of all *in-situ* observations within that grid cell.
4. For all grid cells and dates that did not contain *in-situ* data, we performed the following:
  - We identified the Hydrologic Unit Codes (HUC) that specify the watersheds at multiple scales–HUC-8, HUC-6, and HUC-4—associated with each grid.
  - For each watershed scale (e.g. HUC-8, HUC-6, or HUC-4) that contained the grid cell with missing data, we identified the gauges within that watershed that contain data. We calculated the arithmetic mean of the gauges within the watershed as well as the the inverse-distance-weighted arithmetic mean, using the distance between the gauges and the grid centroid.

**Fig. 8:**
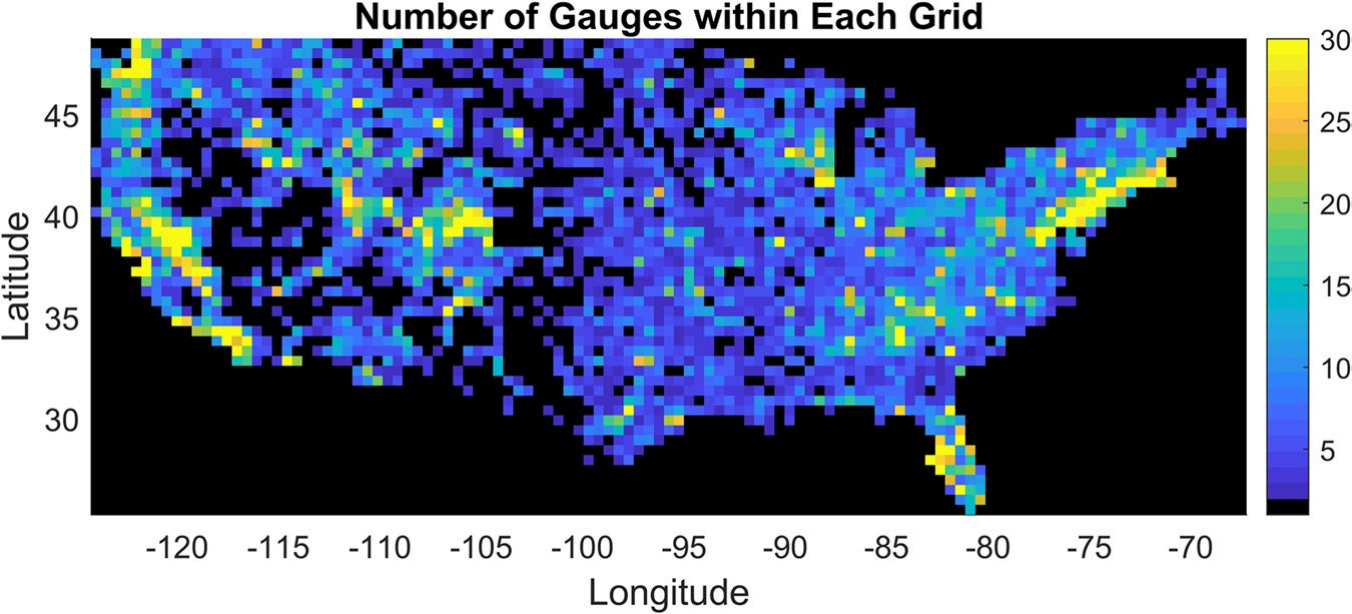
Map of the common grid system showing the number of *in-situ* stream gauges that lie within each grid cell. Grids that are black do not have any *in-situ* gauges within their extent.

Streamflow data obtained in step 1 came from the USGS Water Data for the Nation web interface (https://waterdata.usgs.gov/nwis). Over thirty years of data (1990–2022) were downloaded as text files for all gauges (23,084 total) located in the 18 hydrologic regions within the CONUS. Figure 8 shows the number of gauges that lie within each grid and highlights variations in gauge density across the US. Many of the model grids (13.4%) do not contain a single streamflow gauge, and many of the gauges have significant data gaps within the 2000 to 2022 period of interest. Over this full twenty-two-year period, the average percent of model grids that do not have concurrent gauge data is 25.1%.

**Fig. 9:**
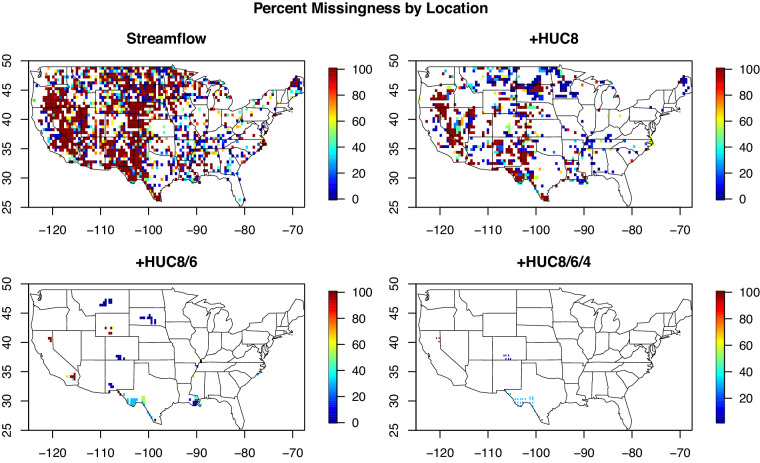
Percent missingness for 7-day average streamflow data, 2000–2022. Only grid cells with some amount of missing data are shown in color. The top left panel shows baseline missingness for USGS streamflow data only. The other three panels infill missing data by using the first available averages from increasingly larger watersheds (HUC8, HUC6, HUC4) containing a grid cell missing any data.

For steps 2 and 3, the daily average streamflow observations from each gauge were converted to 7-day, 14-day, and 28-day averages that align with the Tuesday of each week. If less than half of the daily observations were available over the averaging period, then NA (i.e. null) values were applied. Empirical frequency curves were produced for each gauge and for each averaging period by sorting the thirty-year time series, and then each 7-day, 14-day, and 28-day observation was converted to a percentile based on its position within the sorted data and the total number of valid observations. However, as shown in Figs. 8 and 9, there are many grids and time periods where no data is available within a grid.

**Fig. 10:**
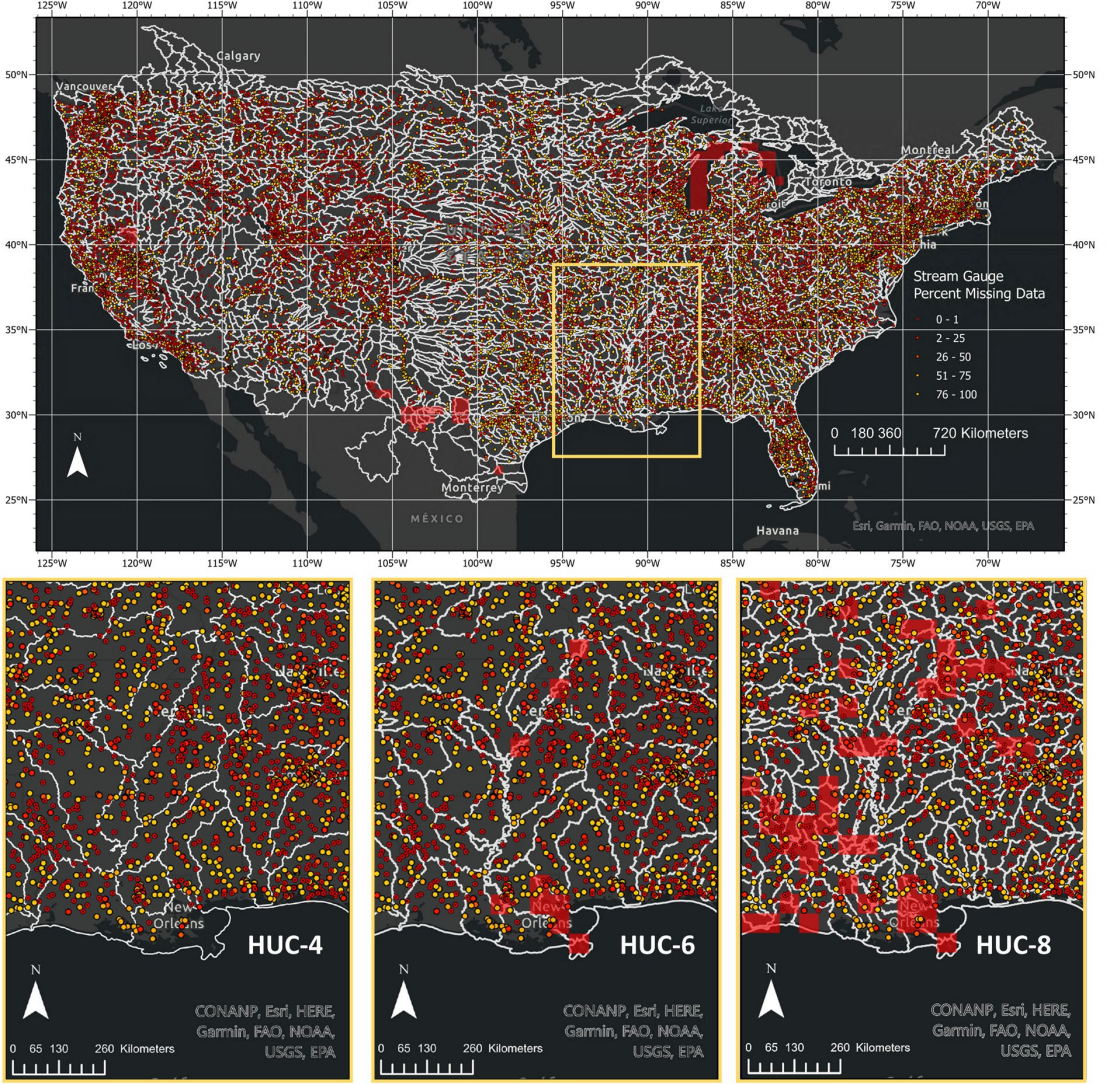
Top image: All *in-situ* stream gauges within the CONUS with HUC-4 watershed boundaries (light gray). The colors of the stream gauges range from red to yellow, indicating the percentage of valid data during the 2000 to 2022 period. Grids that do not have any *in-situ* data at the HUC-4 scale are highlighted in red. Bottom images: Close-up view of the Southern US that shows stream gauges within HUC-4, HUC-6, and HUC-8 watershed boundaries, and highlights (red) grids where *in-situ* data could not be identified within the watershed using the corresponding HUC scale.

Step 4 involves infilling missing grid cells whose streamflow averages were recorded as NA in steps 2 and 3. The simplest approach to filling the data gaps would be to search for gauges that are closest to missing grid values; however, streamflow is largely governed by the physical terrain, and so the measure of “closeness” should match physical terrain. To account for physical flow processes in the gap-filling procedure, the Standardized USGS Hydrologic Unit Code (HUC) watersheds were used to identify the gauges “near” a missing grid value. HUC watersheds are standardized and can be downloaded from the National Map (https://hydro.nationalmap.gov/). They are derived from digital elevation models of the landscape and delineate boundaries of land that drain to the same outlet point. The area within a HUC watershed is therefore hydrologically connected, and the associated streamflow magnitudes should be correlated within. Considering the distance between gauges varies substantially across the CONUS, grid centroids were matched with watersheds at three different scales – HUC-4, HUC-6, and HUC-8. Each of these scales is illustrated in relation to the *in-situ* gauges in Fig. 10.

Gaps within the gridded dataset were subsequently filled using the HUC/gauge match-up information according to the procedure outlined in step 4. Distances between each grid centroid and gauges contained within each watershed were calculated in decimal degrees using the latitude and longitude information associated with each gauge. These distances and flow percentiles were then queried to calculate the inverse distance weighted (IDW) mean, defined as

$${Q}_{avg}=\frac{\mathop{\sum }\limits_{i=1}^{n}{Q}_{i}{d}_{i}^{-1}}{\mathop{\sum }\limits_{i=1}^{n}{d}_{i}^{-1}},$$

where *Q*_*i*_ is the flow percentile for gauge *i*, and *d*_*i*_ is the distance from the grid centroid for gauge *i*. We also separately computed an arithmetic mean for each missing value with no distance weighting. The IDW mean allows more weight to be placed on gauges that are closer to the grid centroid, an approach that is commonly applied to interpolate geospatial data^41–43^ If this gap-filling procedure is limited to the finest (HUC-8) watershed scale, then the average percentage of missing values drops from 25.1% to 15.6% (based on the 7-day flows). If the HUC-6 and HUC-4 watershed scales are used to search for eligible gauges, then the average percentage of missing values drops further to 1.8% and 1.2%, respectively. The grids that do not have fill values at each of the watershed scales are highlighted in Fig. 9. At the coarsest scale, the only remaining missing grids are located in arid locations, including Southwest Texas along the border with Mexico, a small watershed that lies between the central California and Nevada border, a small region of Southern Colorado.

### Teleconnections

**Fig. 11:**
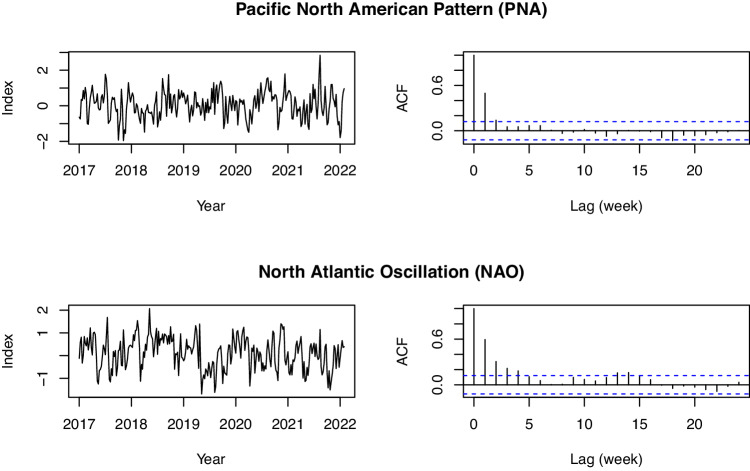
Left panels: Raw data for the two teleconnections from 2017–2022. Right panel: Autocorrelation functions of the two teleconnections.

**Fig. 12:**
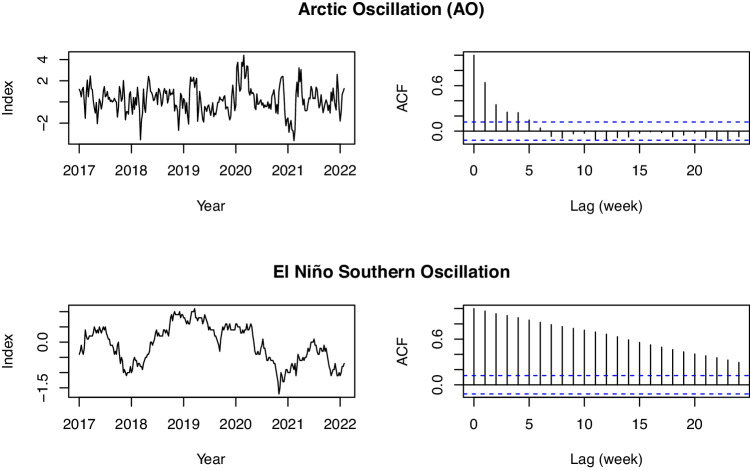
Left panels: Raw data for the two teleconnections from 2017–2022. Right panel: Autocorrelation functions of the two teleconnections.

Teleconnections data were obtained for four commonly used indices: Niño 3.4 (ENSO), the Pacific/ North American Pattern (PNA), the Arctic Oscillation (AO), and the North Atlantic Oscillation (NAO). These were selected from those available at National Weather Service Climate Prediction Center (https://www.cpc.ncep.noaa.gov/). These four teleconnections indices are not spatially varying, so their values in these data do not differ by grid cell but only time. PNA, NAO, and AO data are available daily covering the entire study period at the National Weather Service Climate Prediction Center. We took 7-day running averages of each index that are reported on the Tuesday of each week to match the USDM data and align with the NLDAS-2 and streamflow data. Thus, dates are 1/4/2000, 1/11/2000, etc. ENSO was not available at a daily timescale, but was available at a weekly timescale (https://www.cpc.ncep.noaa.gov/data/indices/wksst9120.for). The date of each weekly index is only one day later that the USDM (so, 1/5/2000, 1/12/2000, etc.). Given high temporal autocorrelation of ENSO (see Fig. 12), we disregarded this mismatch by a single day, and attributed each weekly index value to the prior day to match the temporal support of all other variables in the data. Figures 11 and 12 show five years of each of the raw teleconnections indices, as well as autocorrelation functions for each, which are defined as

$${\gamma }_{k}=\frac{Cov({X}_{t},{X}_{t+k})}{Var({X}_{t})},$$

where *k* = 1, 2, … defines the length of the weekly time lag for variable *X*. Given the prominence of ENSO in climate research in North America, we also provide the 14-day, 28-day and 84-day averages of ENSO to allow the user to consider longer time frames.

## Data Records

**Fig. 13:**
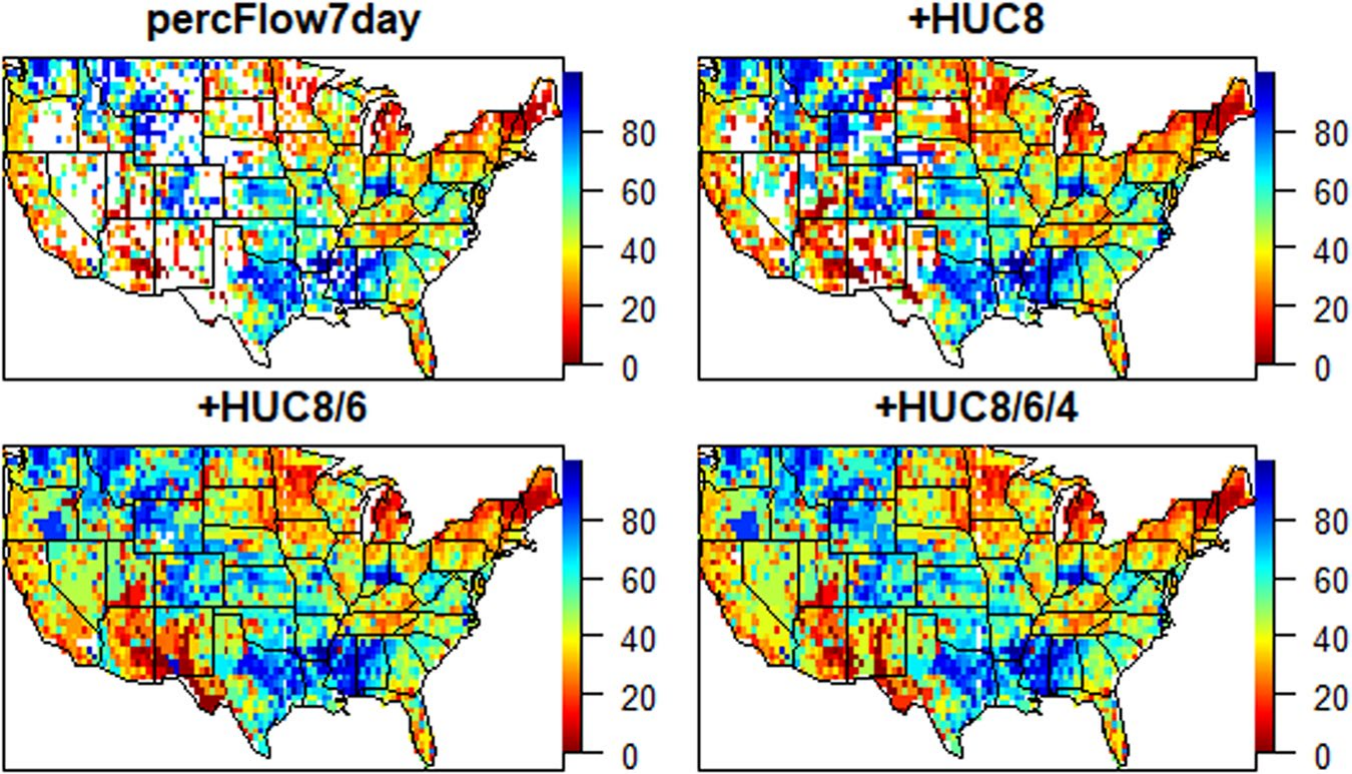
Example streamflow data from the week of June 22, 2021, which is the same date shown in Fig. 6. The top left panel shows the single variable percFlow7day, the percentage of streamflow over 7 days for all gauges within each 0.5 degree grid cell. Since some grid cells have no gauges, these are missing and showin in white in the top left figure. The next three successive panels fill in missing values using values averaged over HUC8 watersheds (avg.HUC8.7day), both HUC8 and HUC6 watersheds (avg.HUC8.7day and avg.HUC6.7day), and HUC8, HUC6 and HUC4 watersheds (avg.HUC8.7day, avg.HUC6.7day and avg.HUC4.7day).

**Fig. 14:**
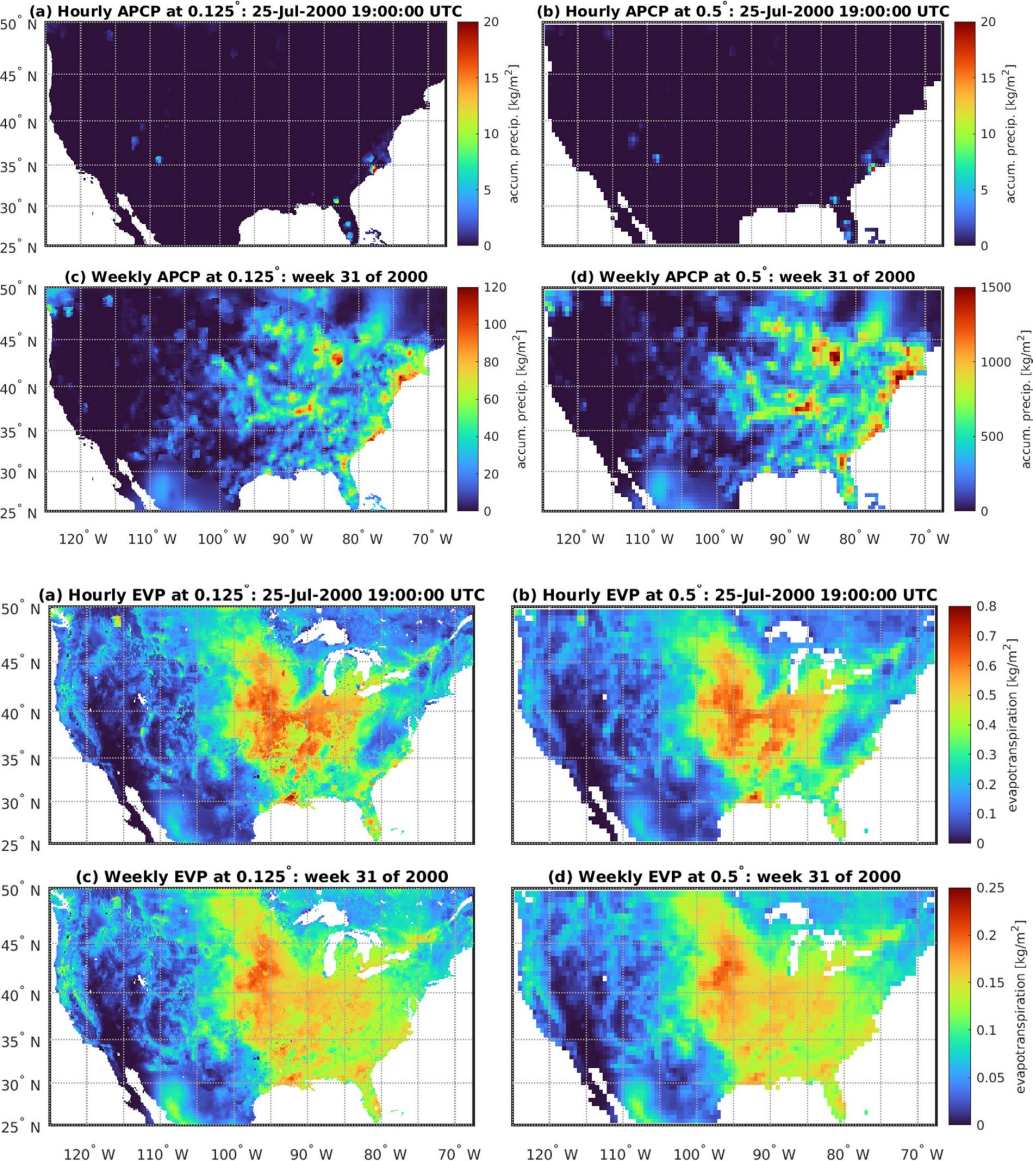
Comparison of raw and upscaled data for APCP (top 4 images) and EVP (bottom 4 images). For each variable, images show: (**a**) raw NLDAS-2 data at the hourly and 0.125 degree resolution; (**b**) upscaled hourly data at 0.5 deg resolution; (**c**) upscaled weekly data at the 0.125 degree resolution; and (**d**) upscaled weekly data at the 0.5 degree resolution. A threshold of 70% land pixels is used at the native resolution to define a land pixel at the upscaled resolution. White areas indicate water pixels.

**Fig. 15:**
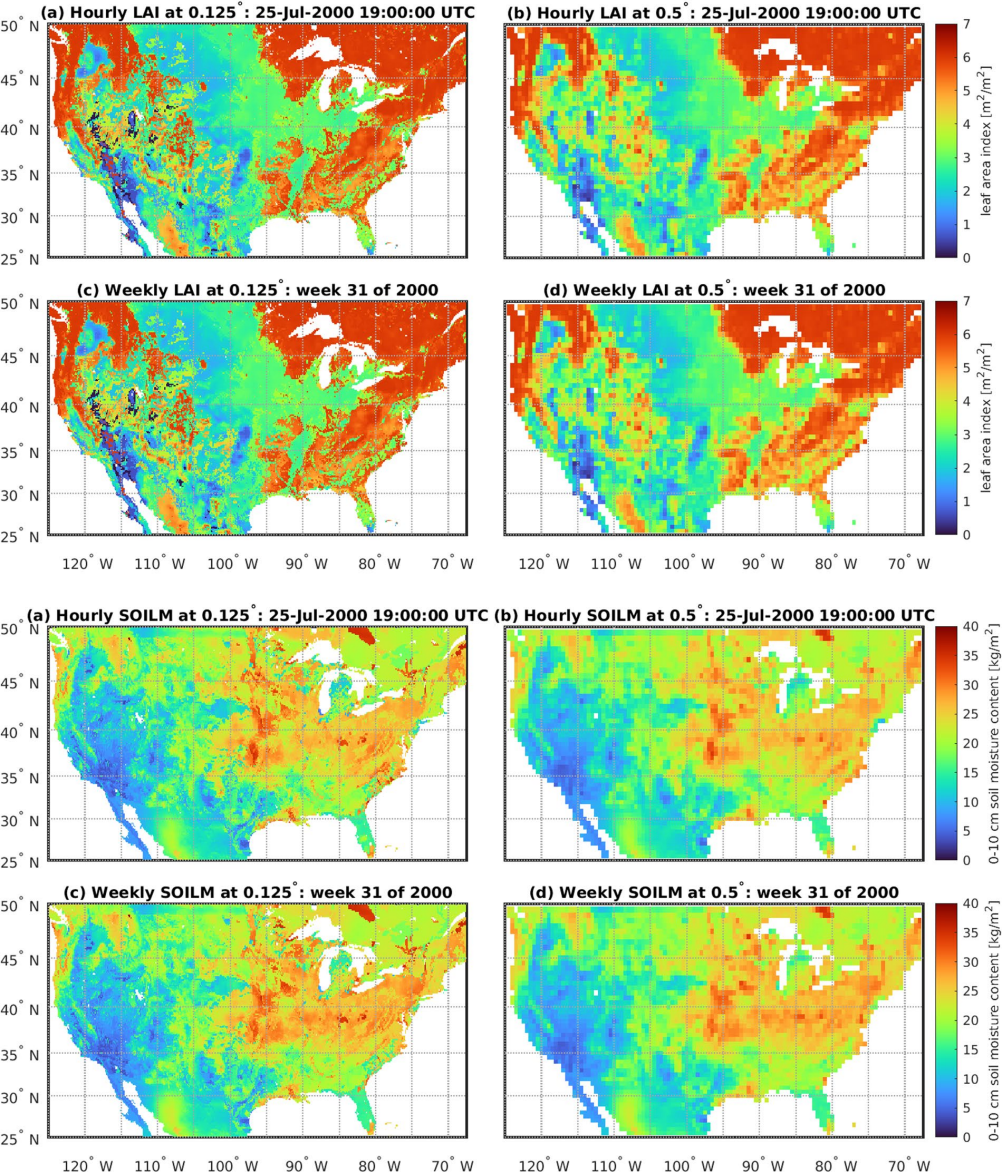
Comparison of raw and upscaled data for LAI (top 4 images) and SOILM (bottom 4 images). For each variable, images show: (**a**) raw NLDAS-2 data at the hourly and 0.125 degree resolution; (**b**) upscaled hourly data at 0.5 deg resolution; (**c**) upscaled weekly data at the 0.125 degree resolution; and (**d**) upscaled weekly data at the 0.5 degree resolution. A threshold of 70% land pixels is used at the native resolution to define a land pixel at the upscaled resolution. White areas indicate water pixels.

The resulting data product is available on DataDryad at 10.5061/dryad.g1jwstqw7^44^. These data are stored in a flat csv file, with 45 columns whose names and descriptions are shown in Table 3. This dataset has one row for each grid/time combination taken across all unique 3258 grid cells and 1174 time periods. Sample maps of many variables are shown in Figs. 6, 13, 14 and 15.

**Table 3:** Descriptions of the 45 variables in the drought data product.

Variable Name	Description
time	Time of the record in YYYYMMDD format
grid	Alphanumeric unique ID given to each grid cell
lon	Longitude of the grid centroid, in degrees
lat	Latitude of the grid centroid, in degrees
drought	Ordinal value of drought, with 0, D0, D1, D2, D3, and D4 as possible values
apcp	Accumulated precipitation
evp	Total evapotranspiration
pevap	Potential evapotranspiration
pevpr	Potential Latent Heat Flux
soilm	Soil Moisture
ssrun	Soil Runoff
tsoil	Soil Temperature
lai	Leaf Area Index
snod	Snow Depth
snom	Snow Melt
snowc	Snow Cover Fraction
weasd	Water Equivalent of Accumulated Snow Depth
percFlow7day	7-day average streamflow percentile. NA if missing.
percFlow14day	14-day average streamflow percentile. NA if missing.
percFlow28day	28-day average streamflow percentile. NA if missing.
avg.HUC8.7day	Average of 7-day average streamflow values taken over all gauges in the HUC 8 watershed containing the grid cell. Only computed if percFlow7day is NA.
avgDist.HUC8.7day	Inverse distance-weighted average of 7-day average streamflow values taken over all gauges in the HUC 8 watershed containing the grid cell. Only computed if percFlow7day is NA.
avg.HUC6.7day	Average of 7-day average streamflow values taken over all gauges in the HUC 6 watershed containing the grid cell. Only computed if both percFlow7day and avg.HUC8.7day are NA.
avgDist.HUC6.7day	Inverse distance-weighted average of 7-day average streamflow values taken over all gauges in the HUC 8 watershed containing the grid cell. Only computed if both percFlow7day and avg.HUC8.7day are NA.
avg.HUC4.7day	Average of 7-day average streamflow values taken over all gauges in the HUC 4 watershed containing the grid cell. Only computed if percFlow7day, avg.HUC8.7day and avg.HUC6.7day are all NA.
avgDist.HUC4.7day	Inverse distance-weighted average of 7-day average streamflow values taken over all gauges in the HUC 4 watershed containing the grid cell. Only computed if percFlow7day, avg.HUC8.7day and avg.HUC6.7day are all NA.
avg.HUC8.14day	Analagous to the six variables listed above, but computed using 14-day average streamflow.
avgDist.HUC8.14day	
avg.HUC6.14day	
avgDist.HUC6.14day	
avg.HUC4.14day	
avgDist.HUC4.14day	
avg.HUC8.28day	Aanalagous to the six variables listed above, but computed using 28-day average streamflow.
avgDist.HUC8.28day	
avg.HUC6.28day	
avgDist.HUC6.28day	
avg.HUC4.28day	
avgDist.HUC4.28day	
pna	Pacific North American pattern
nao	North Atlantic Oscillation
ao	Arctic Oscillation
enso, enso14, enso28, enso84	El Niño Southern Oscillation at 7-day, 14-day, 28-day, and 84-day averages

NAs are used to indicate any missing or undefined entries throughout, and different variables have different patterns of missingness. In the NLDAS-2 data files, data representing land surface processes were labeled as undefined if they occurred over water. In the upscaled dataset, grid cell R25 at (−112.75, 41.25) covers Great Salt Lake and contains less than 70% land, so it was accordingly removed from the full dataset. There are no missing values for drought. Missing data for the streamflow variables is shown in Fig. 9 and described in detail in the previous section. No teleconnections values are missing for any time period.

## Technical Validation

### Upscaling of NLDAS-2 Land Surface Variables

We investigate the variables of evapotranspiration (EVP), leaf area index (LAI), precipitation (APCP), and soil moisture (SOILM) to validate the spatial and temporal upscaling of the NLDAS-2 land surface data from its native 0.125° spatial and hourly temporal resolution to the spatially and temporally aggregated weekly grid data. Figures 14 and 15 demonstrate the loss in heterogeneity reflecting complexity in the land surface when upscaling from hourly to weekly (top row to bottom for each variable) as well as 0.125 degrees to 0.5 degrees (left column to right column for each variable). The consequences of this loss of heterogeneity can be minimal if analyses and conclusions are restricted to the coarser weekly and 0.5 degree resolution. Larger regional patterns of low evaporation rates over arid regions of the US and high rates over humid regions remain.

**Fig. 16:**
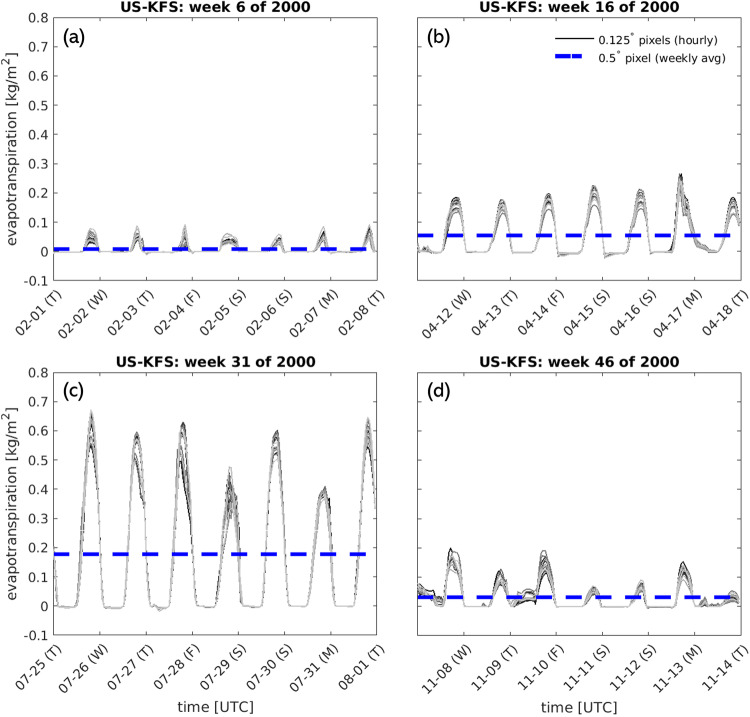
Comparison of time series of raw EVP data at 0.125 deg data to upscaled 0.5 deg data for a single pixel representing the Kansas Field Station (39.0561°, −95.1907°) during: (**a**) winter, (**b**) spring, (**c**) summer, and (**d**) fall. All 16 pixels at the native spatial resolution that contribute to the upscaled 0.5 deg grid cell are shown as individual gray lines. The weekly average for the 0.5 deg grid cell is shown as a dashed blue line.

**Fig. 17:**
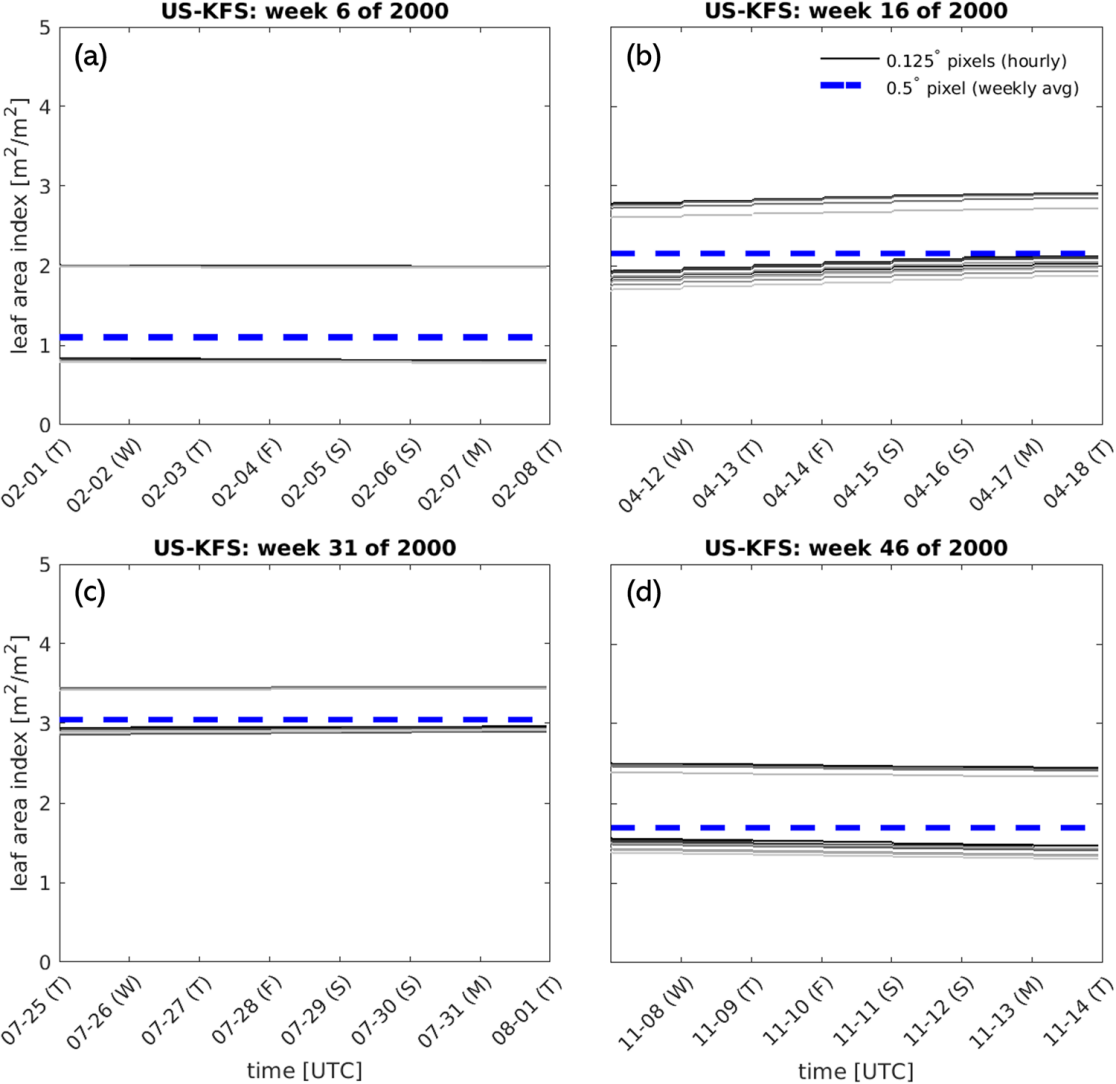
Comparison of time series of raw LAI data at 0.125 deg data to upscaled 0.5 deg data for a single pixel representing the Kansas Field Station (39.0561°, −95.1907°) during: (**a**) winter, (**b**) spring, (**c**) summer, and (**d**) fall. All 16 pixels at the native spatial resolution that contribute to the upscaled 0.5 deg grid cell are shown as individual gray lines. The weekly average for the 0.5 deg grid cell is shown as a dashed blue line.

**Fig. 18:**
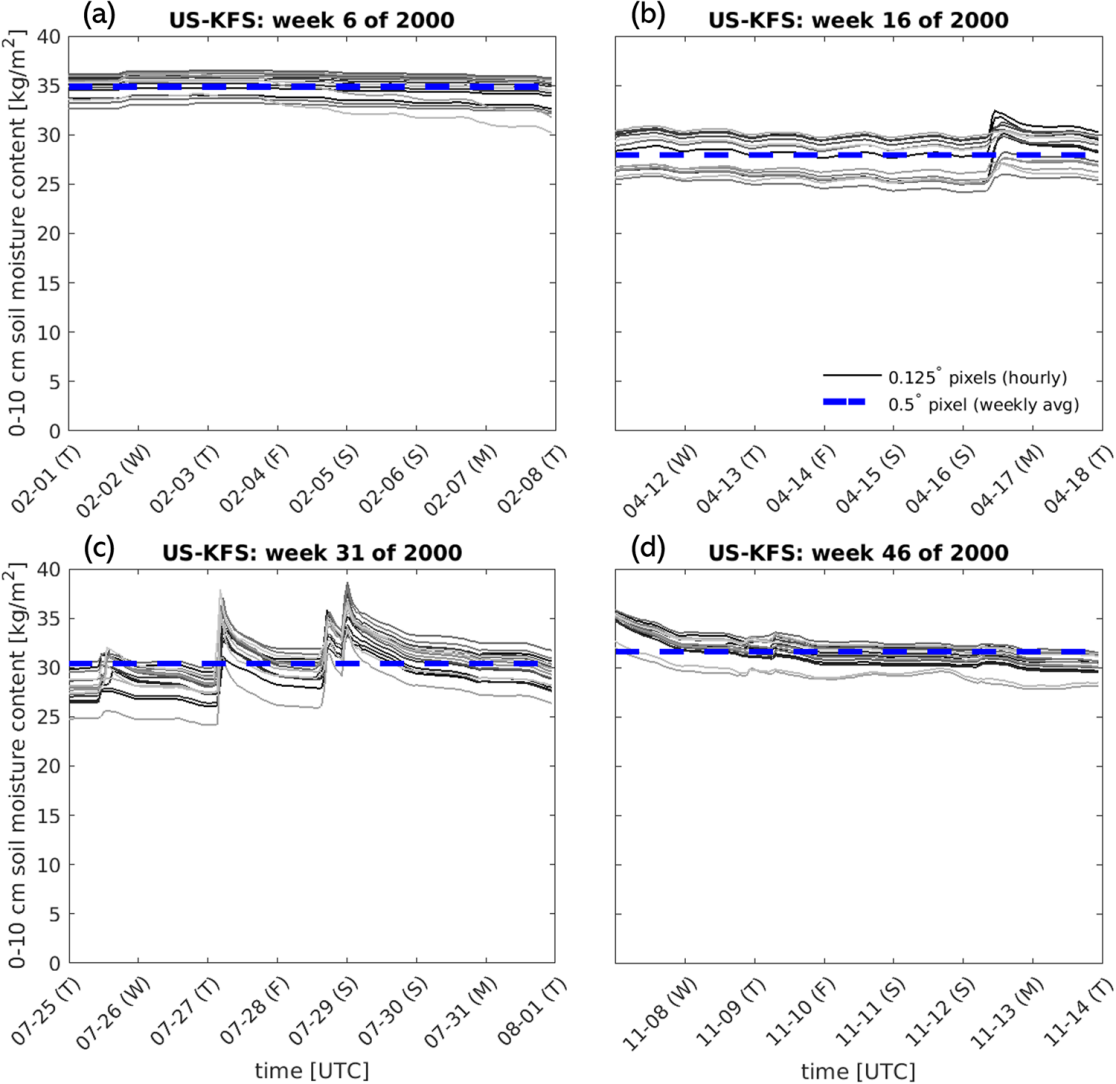
Comparison of time series of raw SOILM data at 0.125 deg data to upscaled 0.5 deg data for a single pixel representing the Kansas Field Station (39.0561°, −95.1907°) during: (**a**) winter, (**b**) spring, (**c**) summer, and (**d**) fall. All 16 pixels at the native spatial resolution that contribute to the upscaled 0.5 deg grid cell are shown as individual gray lines. The weekly average for the 0.5 deg grid cell is shown as a dashed blue line.

Aggregating the data in time from hourly amounts to weekly averages removes the diurnal variability for some variables (i.e. EVP) that are strongly controlled by incoming solar radiation (Fig. 16). However, the weekly averages follow the expected seasonal variability and are higher in magnitude during the summer, when daily maximum evapotranspiration is high, and near zero in the winter when little energy is available for evapotranspiration to occur (Fig. 16 a,c). For other variables which do not follow diurnal variability, upscaling can aggregate roughly stationary time series values across space (Fig. 17), or smooth over minor temporal variability in variables with relatively low spatial variation at 0.125 degrees (Fig. 18).

### Streamflow

**Fig. 19:**
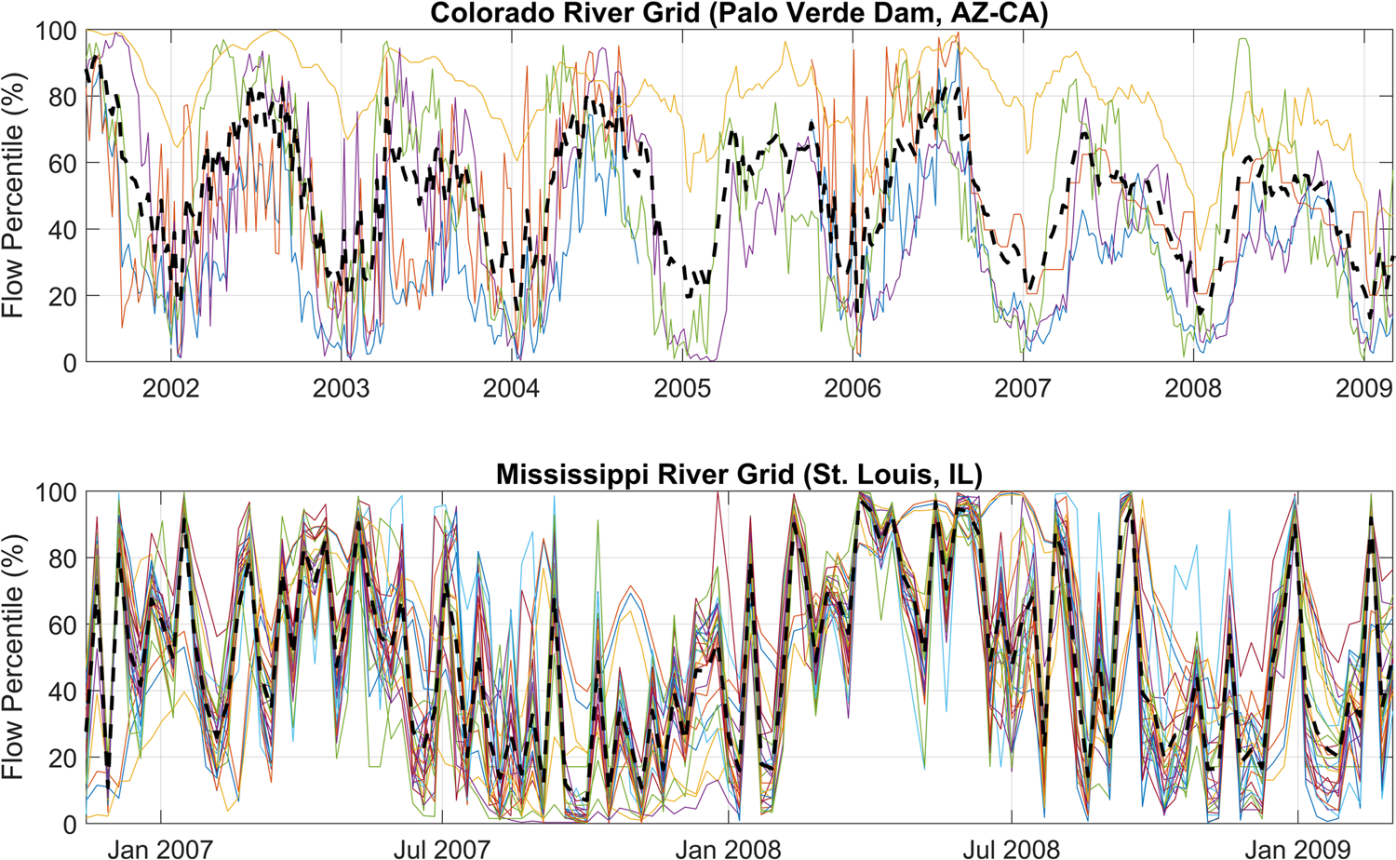
Top image: streamflow percentiles within a grid that contains a gauge on the Colorado River. Individual gauges are represented with the thin solid colored lines, and the grid mean is represented with the thick dashed black line. Bottom: streamflow percentiles within a grid that contains a gauge on the Mississippi River. The time periods were selected to enhance visibility of the plots.

To validate the point-to-grid conversion of the streamflow percentiles, we explored time series of individual grids and quantified the variability between the gridded values and individual gauges that were used to estimate the grid mean. The goal of these analyses was to check that the temporal variations of the grid means are similar to those of the individual gauges, and to assess the variability of the gauge values that were used for gridded estimates. Time series of the 7-day streamflow percentiles for two grids are shown in Fig. 19. The top image shows the values for a grid that overlays the Colorado River near the Palo Verde Dam along the Arizona and California border (gauge site ID 9429100) that contains 5 gauges with valid data. The bottom image shows the values for a grid that overlays the Mississippi River near St. Louis, IL (gauge site ID 7010000) that contains 33 gauges with valid data.

**Fig. 20:**
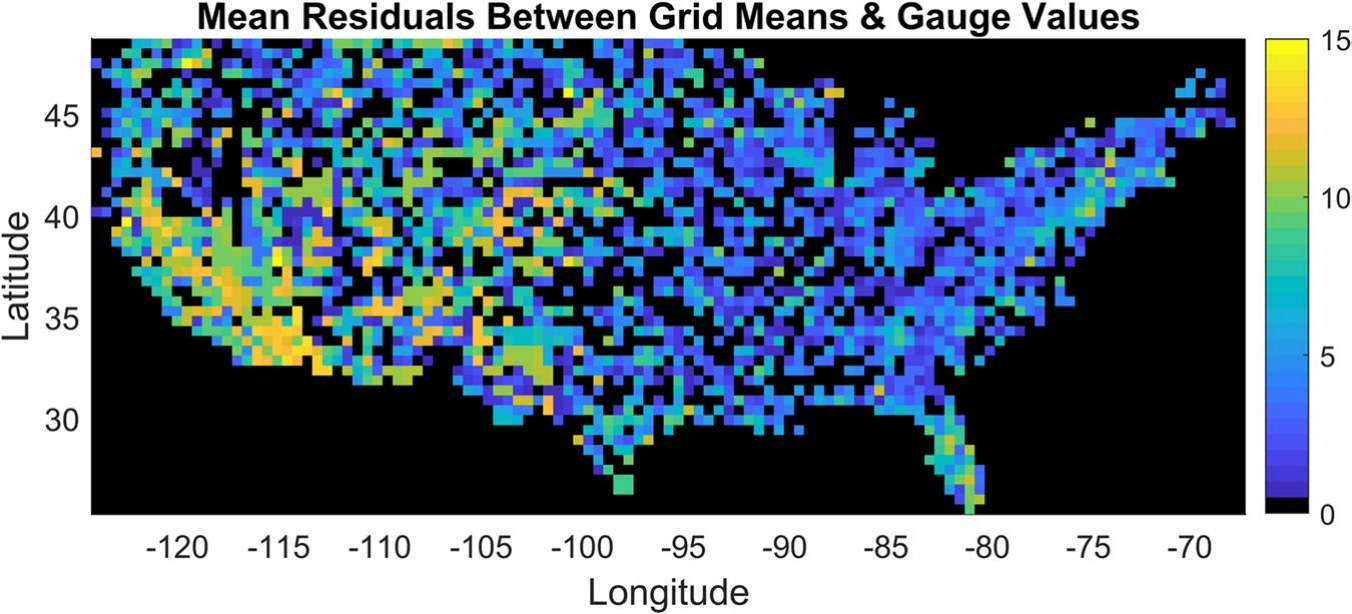
Mean standard deviation of residuals between gauage data and grid cell average.

The variability between gauge values for all grids in the CONUS was explored by calculating the mean of the standard deviation of the residuals, where the residuals are the differences between the averaged grid value and each gauge that was used to estimate the grid value. The 7-day flow percentile values were used in this exercise. These calculations result in a single value for each grid that represents the mean variability over time, which can be presented as a gauge data variability map, shown in Fig. 20. If a grid contains a value of zero, then a single gauge was used to calculate the grid mean.

**Fig. 21:**
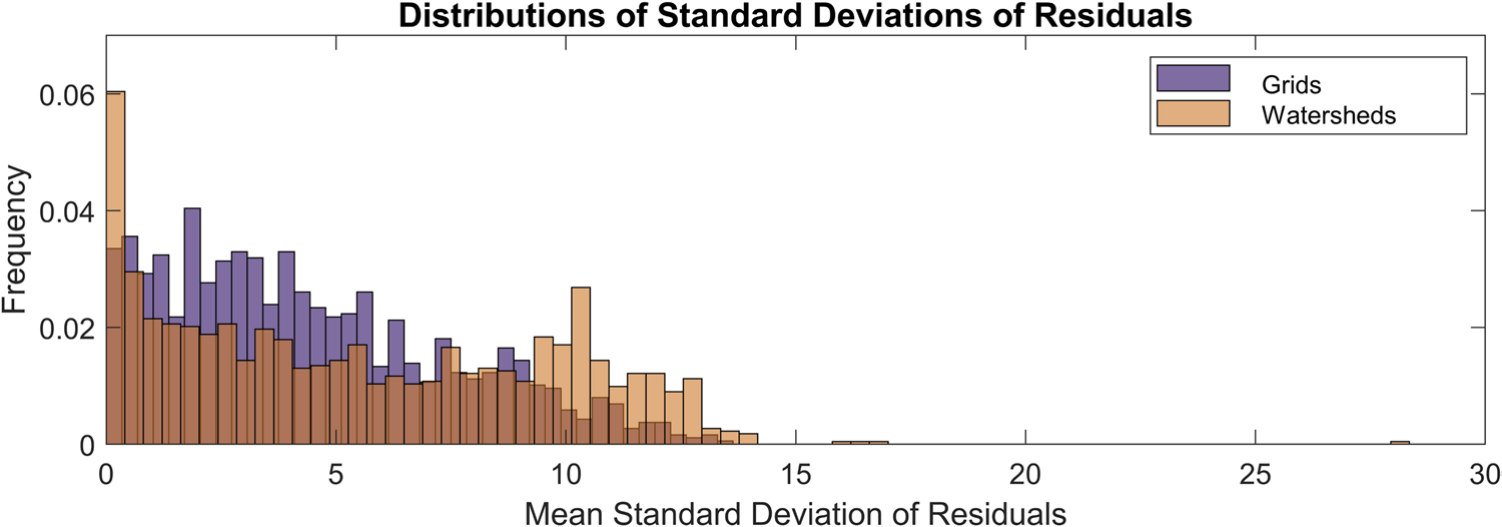
Histogram plots that represent the distribution of the gridded streamflow variability measure. The grids that contain data within their extents are compared to the grids that used the HUC watershed extents to identify gauges.

We expected that the data variability would be greater for grids that used the HUC watershed extents as opposed to those that contained *in-situ* data within its extents. This hypothesis was tested by splitting the mean residuals between these two grid types and plotting the resulting distributions, shown in Fig. 21. The mean standard deviation of residuals for the grids that contain gauges within the extents is 4.37%, and 5.54% for the grids that used the HUC watershed extents. The distribution of this metric is somewhat bi-modal for the grids that were filled using the HUC watersheds, with peaks close to 0% and 10%. The data variability is slightly greater for grids that used the watershed-based filling procedure. However, the overall variability across the CONUS is low, with the exception of a few grids that have values between 15% to 30%. This result and the time series plots suggest that the point-to-grid conversion was performed correctly and that the representative grid values are capturing the multi-temporal behavior of the individual gauges.

To conceptualize this variability measure in the context of streamflow magnitudes in major US rivers, it was applied to the gauge data from the Colorado and Mississippi Rivers, shown in Fig. 19. The mean of the residuals for the grid corresponding with the Colorado gauge is 9.75%, on the high end of the distribution. The mean streamflow recorded by this gauge from 2000 to 2021 was 7,200 cfs, so conceptually the 9.75% variability introduces an uncertainty of about 700 cfs, on average. Similarly, the mean flow of the Mississippi River from 2000 to 2021 at St. Louis, IL was 234,418 cfs, and the mean of the residuals is 8.75%, translating to an estimated average uncertainty of 20,512 cfs.

## Usage Notes

### Time series models for ordinal drought

These data are indexed in three dimensions—space (grid cells with centroids indexed by latitude and longitude) as well as time. If one considers a single fixed grid cell, the drought status at that location can be represented as ${Y}_{t},t=1,\ldots ,T$ for weekly drought status, where ${Y}_{t}\in 1,\ldots ,6$ representing each of the six USDM drought levels. All covariates measured at the same location are ${{\bf{X}}}_{t}={({X}_{1},\ldots ,{X}_{j})}_{t},t=1,\ldots ,T$. This setup permits time series modeling of ordinal drought level in terms of all previous drought history and all previous covariate history. If we define a vector **W**_*t*_ which contains functions of some subset of prior response values ${Y}_{t-1},{Y}_{t-2},\ldots$ and covariates values known through time $t,({{\bf{X}}}_{t},{{\bf{X}}}_{t-1},\ldots )$, one can set up time series models for ordinal data such as the cumulative odds time series model^45^,

$$\log \left(\frac{P({Y}_{t}\le j)}{P({Y}_{t} > j)}\right)={\beta }_{0,j}+{\bf{W}}\beta ,j=1,\ldots ,J.$$

One could also choose the standard normal cumulative distribution function Φ(·) to yield a familiar probit link between cumulative probabilities and covariates as

$${\rm{probit}}(P({Y}_{t}\le j))={\beta }_{0,j}+{\bf{W}}\beta ,j=1,\ldots ,J.$$

These time series models could be used to forecast future ordinal drought levels with full accounting of uncertainty. The R package ordinal can be used to fit cumulative link models for ordinal regression with both fixed and random effects^46^. For more detail on fitting such models, see Fokianos *et al*.^47^. or Weiss *et al*.^48^.

As an extension, one can also introduce a latent continuous variable ${Z}_{t}={\mu }_{t}+{\varepsilon }_{t}$, where *ε*_*t*_ is a sequence of independent and identically distributed random variables with continuous cumulative distribution function *F*, and *μ*_*t*_ can be parameterized in terms of explanatory variables, random effects, and past values of the process ${Z}_{t-1},{Z}_{t-2},\ldots$ Partitioning the support of the latent variable with cutoffs $-\infty ={\alpha }_{0} < {\alpha }_{1} < \cdots  < {\alpha }_{J-1} < {\alpha }_{J}=\infty$ can facilitate modeling the ordinal data. One can recover the observed ordinal variable as

$${Y}_{t}=\mathop{\sum }\limits_{\,j=1}^{J}j\cdot I({\alpha }_{j-1} < {Z}_{t}\le {\alpha }_{j}),$$

where *I*(·) denotes the indicator function, and *j* indexes the *J* possible ordered categories of drought. Marginalizing over *Z*, some of these models are identical to the cumulative odds or probit models described above. However this latent variable formulation also permits a wider class of time series models and can have computational advantages with model fitting^49^.

### Computationally efficient spatio-temporal drought modeling

The prepared data are also indexed by latitude and longitude and therefore hold spatial dependence in addition to temporal dependence described above. Statistical modeling of such spatio-temporal ordinal data is challenging due to the computational expense required with a large number of locations and/or time periods. Feng *et al*.^50^. proposed a composite likelihood estimate approach as one way to address this computational expense, and a spatial-only model of ordinal data which was implemented in the clespr package in R^50,51^. Brewer *et al*. (2014) and Higgs and Hoeting^52^ implemented Bayesian versions of the spatial ordinal model^53^, which can require a large computational cost to achieve mixing. Schliep and Hoeting^54^ proposed a data augmentation approach to improve the performance of MCMC algorithms for fitting Bayesian versions of spatial ordinal models^54^. Schliep *et al*. (2016) extended these models to the spatio-temporal ordinal data setting^49^.

Each of the approaches mentioned above is limited by the computational complexity of spatio-temporal ordinal data, but this can be partially reduced through a common spatial support and parsimonous statistical modeling. In this data set, all variables have been discretized to the same spatio-temporal grid which enables more parsimonious statistical modeling. While change of support methods exist for situations where the response and explanatory variables are misaligned, these approaches add computational complexity. Spatio-temporal statistical modeling of this full data set is already computationally challenging due to the size of the data, so it is advantageous to work with aligned data.

The aforementioned approaches to modeling spatial or spatio-temporal ordinal data can be implemented for continuous space, or geostatistical data, and also for discrete space, or areal data. Our final gridded data set contains the latitude/longitude coordinates of the grid centroids. However, these data are suited for areal spatial modeling techniques and not geostatistical modeling as the values have been processed as previously described so that the value is a representation of the variable across the entire grid cell and not the measured value at the exact point. Areal spatial models, such as conditional autoregressive models or simultaneous autoregressive models, require the specification of a neighborhood weight matrix, or a known matrix that assigns a numeric value to all pairs of locations^55^. A common choice for the weight matrix is to assign the value of 1 if two areal units share a border and a value of 0 otherwise, thus indicating whether or not two locations are “neighbors.” One attractive feature of areal models is that the precision matrix is sparse, which significantly eases computational expense generally associated with large spatial data sets. More specifically, statistical inference with a geostatistical spatio-temporal model has computational complexity of ${\mathcal{O}}({N}^{3}T),$ where *N* is the number of spatial locations and *T* the time periods; whereas approaches based on first-order Markov random fields, such as the conditional autoregressive model, require ${\mathcal{O}}({N}^{3/2}T),$ operations^56^.

### Alternative drought classification

While the US Drought Monitor is a widely-used definition of drought, there remains an active stream of research into refining the definition of drought. In recent work^11^, Hobeichi *et al*. used a set of climate phenomena mostly measured at 0.5 degrees in a random forest algorithm to classify a location and time period as being in drought or no drought. Labels for drought were based on the Drought Impacts Reporter (DIR), a database of drought impacts housed at the U.S. National Drought Mitigation Center^57^. The resulting random forest method for classification was tested on out-of-sample data, and showed comparatively strong statistical performance scores as compared to other commonly used classifiers. These data provided here could be used in a similar fashion to produce labels for drought that differ from the USDM.

### Other possible uses

One could consider the construction of clusters of locations based on similar drought profiles, using techniques such as k-means clustering, hierarchical clustering, fitting a finite mixture model for probabilistic clustering, and other techniques. Such methods could define zones or regions of similar drought experience. These spatio-temporal data are also a collection of 4-way tensors (across lat/lon/time/type), and the decomposition and analysis of such data is an active area of research^58^. These data could serve as a valuable test case for tensor algorithmic development and interpretability.

The gridded representation of measured streamflow is a novel product that offers new opportunities to explore relationships and trends on the CONUS scale. The USGS monitors streamflow trends for select gauges (https://iwaas.wim.usgs.gov/sw-flow-trends/) that are often spaced far apart, and individual studies analyze regional-scale trends^59,60^. However, this new streamflow product enables comprehensive and computationally efficient trend analyses. Furthermore, streamflow can now be easily compared to the other drought-related covariates using the common grid structure, creating opportunities to complete multivariate analyses that can help explain relationships and their variability in time and space. This synoptic set of covariates could also be grouped by their association with different types of drought (meteorological, hydrological, and agricultural) and combined with the gridded USDM to explore the relative influence each drought type on the lumped drought severity index.

Drought is known to affect water supplies, energy production, public health, agriculture, and wildfire potential. Therefore, this new synchronized dataset can be combined with satellite-based observations of fires, such as the MODIS burned area^61^ and active fire^62^ products, to develop improved approaches to fire risk assessments. A primary application of the USDM is to assess risks to croplands and help farmers obtain financial support during drought period. The US Department of Agriculture provides cropland datasets in raster formats (https://croplandcros.scinet.usda.gov/) that can be combined with this new product to study the impact of drought on agricultural productivity, and identify the key hydrological and meteorological drivers of cropland successes and failures across space and time.

There is increasing interest in the relationship between the environment and public health outcomes. The National Center for Environmental Health has noted that drought has both short-term and long-term health effects related to, e.g. the impact on air quality and increased incidence of illness and disease https://www.cdc.gov/nceh/features/drought/index.html. Berman *et al*.^63^ used data from the USDM for counties in the western United States and found that high severity worsening drought was associated with increased risk of respiratory-related mortality among adults ages 65 and older on Medicare^63^. Paull *et al*.^64^ found the drought was the primary climatic driver of increased rates of West Nile Virus^64^. Head *et al*.^65^ found that drought was associated with increased transmission of Valley Fever^65^. This data set can enable further research on the relationship between drought and its related variables and public health outcomes.

## Data Availability

The data can be found at https://datadryad.org/stash/dataset/doi:10.5061/dryad.g1jwstqw7^44^. All code is freely available at https://github.com/heplersa/USDMdata.
